# The Role of Efflux Pumps in the Transition from Low-Level to Clinical Antibiotic Resistance

**DOI:** 10.3390/antibiotics9120855

**Published:** 2020-11-30

**Authors:** Anna Elisabeth Ebbensgaard, Anders Løbner-Olesen, Jakob Frimodt-Møller

**Affiliations:** Center for Peptide-Based Antibiotics, Department of Biology, University of Copenhagen, 2200 Copenhagen, Denmark; anna.ebbensgaard@bio.ku.dk (A.E.E.); lobner@bio.ku.dk (A.L.-O.)

**Keywords:** low-level antibiotic resistance, clinical resistance, efflux pump, efflux pump inhibitors

## Abstract

Antibiotic resistance is on the rise and has become one of the biggest public health challenges of our time. Bacteria are able to adapt to the selective pressure exerted by antibiotics in numerous ways, including the (over)expression of efflux pumps, which represents an ancient bacterial defense mechanism. Several studies show that overexpression of efflux pumps rarely provides clinical resistance but contributes to a low-level resistance, which allows the bacteria to persist at the infection site. Furthermore, recent studies show that efflux pumps, apart from pumping out toxic substances, are also linked to persister formation and increased spontaneous mutation rates, both of which could aid persistence at the infection site. Surviving at the infection site provides the low-level-resistant population an opportunity to evolve by acquiring secondary mutations in antibiotic target genes, resulting in clinical resistance to the treating antibiotic. Thus, this emphasizes the importance and challenge for clinicians to be able to monitor overexpression of efflux pumps before low-level resistance develops to clinical resistance. One possible treatment option could be an efflux pump-targeted approach using efflux pump inhibitors.

## 1. Introduction

Antibiotic resistance is one of the biggest public health challenges of our time. During the “golden era” of antibiotic discovery (e.g., 1940s through 1960s), effective novel antibiotics were frequently discovered and introduced to the market. However, the current global antibiotic resistance crisis is a result of massive antibiotic consumption both in the clinic and in agriculture along with a progressively declining introduction of novel antibiotics [1,2]. The pace of development of novel antibiotics is slow, mainly due to the huge cost of (large) clinical trials. Furthermore, even if an antibiotic is successfully introduced to the market, its use in the clinic will drop as resistance to it inevitably develops. Thus, novel antibiotic research and development projects will, on average, take a sizeable loss, why most pharmaceutical companies peruse other avenues [3].

The bacterial cell envelope has the ability to prevent the entry of antibiotics into the cell (“permeability barrier”); however, it is increasingly recognized that the function of efflux pumps, acting either alone or along with decreased expression of porins, constitutes parts of this “barrier” [4]. Even so, decreased entry of drugs into the bacterial cell is still an important factor to be considered. Genes encoding efflux pumps are found on both plasmids (e.g., transmissible elements) or on the chromosome. In this review, we will focus on the chromosomally encoded efflux pumps.

## 2. Physiological Role for Efflux Pumps

Efflux pumps are highly conserved elements that are ubiquitous throughout nature in all types of cells, from prokaryotic to eukaryotic organisms [4]. In bacteria, the role of efflux pumps is broad and has been shown to be important in pathogenicity, bacterial physiology, and metabolism [4]. Apart from enabling the bacteria to survive in the presence of toxins, biocides, heavy metals, and antimicrobial agents, efflux pumps are also reported to play important roles in quorum sensing (by pumping out quorum-sensing signal molecules [5]), adherence, invasion, colonization of host cells [6], and biofilm formation [7]. Here, we focus on the role of efflux pumps in tolerance/resistance to antimicrobials. Some efflux pumps have the capacity to pump out different antimicrobial agents (i.e., multidrug resistance (MDR) efflux pumps). Within the prokaryotic kingdom, six major families of efflux pumps are associated with MDR: the multidrug and toxic compound extrusion (MATE) family [8], the resistance-nodulation-division (RND) family [9], the adenosine triphosphate (ATP)-binding cassette (ABC) superfamily [10], the small multidrug resistance (SMR) family [11], the proteobacterial antimicrobial compound efflux (PACE) family [12], and the major facilitator superfamily (MFS) [13]. While RND [14] and PACE [15] efflux pumps are unique to Gram-negative bacteria, MATE, ABC, SMR, and MFS are found in both Gram-positive and Gram-negative bacteria.

Bacteria are able to adapt quickly to the selective pressure exerted by antimicrobial agents in numerous ways, including (over)expression of efflux pumps, drug target modification, modified cell wall, drug inactivation, and modification. Recently, the World Health Organization (WHO) published a list of antibiotic-resistant pathogens, including both Gram-positive and Gram-negative bacteria, for which new and effective antibiotics are urgently needed [16]. All of these high-priority pathogens have reported antibiotic resistance mediated by efflux pumps. This highlights the impact and importance of efflux pumps in the clinical setting [17]. The expression of efflux pumps are regulated by numerous mechanisms; some are constitutively expressed, thus conferring intrinsic tolerance/resistance to their substrates, while others are only transiently induced by their substrates [4]. Common for both types is that high levels of expression lead to increased efflux of the substrate, which is a result of either the inducer being present or a mutation in the gene downregulating efflux pump expression [18]. Even though the expression of a single efflux pump can confer MDR, simultaneous overexpression of more than one efflux system has been described in clinical isolates of *Stenotrophomonas maltophilia* [19] and *Pseudomonas aeruginosa* [20].

## 3. Low-Level Resistance Can Progress to Full Antibiotic Resistance

**Figure 1:**
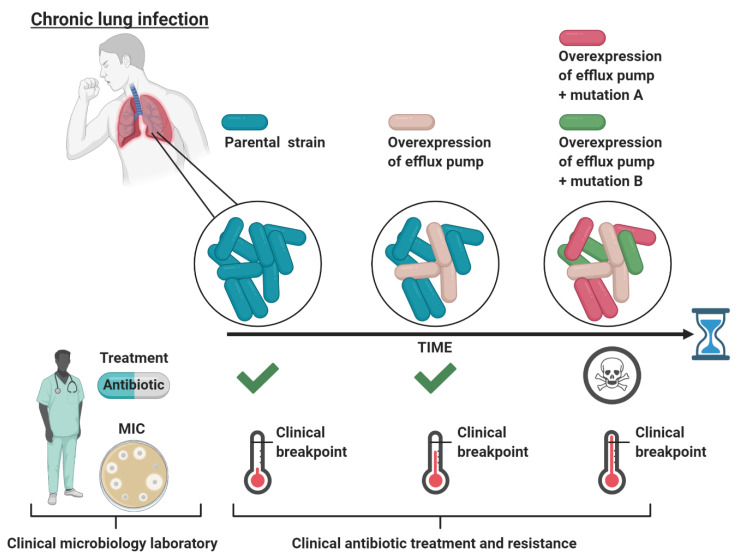
Schematic representation of the evolutionary path of bacteria in chronic lung infections during continuous antibiotic treatment. Initially, the bacteria causing the infection are sensitive (MIC below the clinical breakpoint) to the treating antibiotic as determined by the MIC at the clinical microbiology laboratory, leading to treatment. Next, the antibiotic does not distribute equally throughout the lungs, such that certain niches only obtain a minimal amount of antibiotic. In these niches, an efflux pump active population conferring low-level clinical resistance can survive and persist. However, when tested at the clinical microbiology laboratory, this efflux pump active population is categorized as sensitive to the treating antibiotic, leading to continuous treatment. Lastly, the low-level-resistant population now has an evolutionary opportunity, while surviving in sublethal antibiotic concentrations, to evolve to high-level resistance through mutations in antibiotic target genes (e.g., mutations A and B). These evolved bacteria are now resistant (MIC above the clinical breakpoint) to the administrated antibiotic, leading to treatment failure for the patient. The figure is created with BioRender.com.

A large body of evidence shows that increased expression of efflux pumps does not necessarily (if rarely) increase the minimal inhibitory concentration (MIC) of many antimicrobials (and other detergent-like substrates) above the clinical breakpoints (reviewed by Piddock [4]). The MIC is defined as the lowest concentration of an antibiotic required to inhibit bacterial growth, whereas the clinical breakpoint defines whether an organism is susceptible or resistant to the antibiotic, as determined by the European Committee on Antimicrobial Susceptibility Testing (EUCAST). A bacterium is considered susceptible to the antibiotic if the MIC is lower or equal to the clinical breakpoint, while it is considered resistant to the antibiotic if the MIC is higher than the breakpoint. The antibiotic dose used to treat a bacterial infection is typically much higher than the MIC determined for the infecting bacteria in the clinical microbiology laboratory [21]. Nevertheless, the effective in vivo antibiotic concentration is likely to be below the MIC in some body niches, thus allowing bacteria with low-level resistance to survive [21]. Thus, in spite of the fact that increased expression of efflux pumps does not provide clinical resistance per se, efflux pump-mediated low-level-resistant bacteria might persist at the infection site, which could present an opportunity to evolve into high-level clinical resistance through the acquisition of additional mutations. This scenario is exemplified in chronic lung infections for *P. aeruginosa* (in cystic fibrosis) [22] (Figure 1).

During antibiotic treatment of cystic fibrosis patients, the in vivo antibiotic concentration in the lungs and sputum might never reach the MIC concentration. For *P. aeruginosa* isolated from young cystic fibrosis patients who were treated with fluoroquinolones, aminoglycosides, macrolides, β-lactams, and the antimicrobial peptide colistin, some of the most frequently mutated genes are found in the negative regulators of RND efflux systems, with *mexZ* (negative regulator of MexXY-OprM) being the most frequent [23]. When competing in clinically relevant sub-MIC growth conditions (using amikacin or ciprofloxacin), *mexZ*-deficient *P. aeruginosa* is shown to outcompete the wild-type strain [22]. A second example comes from *Mycobacterium tuberculosis*, the causative agent of the chronic lung disease tuberculosis. *M. tuberculosis* cells with low-level resistance to isoniazid caused by increased efflux pump activity was found to progress into a high isoniazid resistance by the acquisition of secondary mutations providing a link between efflux pump activity and development of high-level drug-resistance-causing mutations [24].

These findings emphasize the importance and challenge for clinicians to monitor antibiotic tolerance before low-level-resistant bacterial populations potentially become antibiotic resistant [21]. Dewachter et al. [25] recently emphasized that this is an important problem, which should no longer be ignored in the clinical microbiology practice; better diagnostic and therapeutic approaches are urgently needed. Thus, by fast, reliable diagnostic tools, all phenotypes present in the bacterial population could be resolved, which makes it feasible to implement a combinatorial antibacterial therapy, which in theory could include the use of efflux pump inhibitors, targeting and eradicating all phenotypes in the infecting bacterial population [25].

## 4. The Fitness Cost of Efflux Pump Overexpression

Intuitively, upregulation of efflux pumps should lead to increased energy consumption due to the constant extrusion of (toxic) substances. Thus, cells with an increased efflux pump activity will have reduced fitness relative to the parental population in the absence of the efflux pump substrate. Reduced fitness can be restored by compensatory mutation(s), which offsets the cost of upregulated/constitutive active efflux pump(s). If a compensatory mutation is acquired, the mutant population will no longer be outcompeted by the parental population in the absence of the selecting agent [26]. For example, Pacheco et al. [27] reported that in *P. aeruginosa* overexpression of the RND efflux pump, MexEF-OprN does not lead to decreased fitness. Here, *P. aeruginosa* compensates by “metabolic rewiring,” which leads to increased anaerobic and aerobic respiration, which compensates for the fitness costs of overexpression of the efflux pump. This creates a dangerous clinical scenario, where the low-level-resistant strains are able to persist with or without the presence of the antibiotic and, when faced with a higher antibiotic concentration, have a higher probability to give rise to high-level antibiotic resistance.

## 5. Efflux Pumps Are More Than “Just” Efflux

Historically, efflux pumps are associated with MDR; however, two recent efflux pump discoveries indicate a broader functional role of efflux pumps beyond the transport of toxic compounds. Thus, efflux pumps could be involved in antibiotic survival by mechanisms that may be unrelated to efflux. Persister cells are a small fraction within a bacterial population, which are able to survive antibiotic treatment. These are phenotypically distinct from antibiotic-resistant bacterial cells, and survival in the presence of antibiotics seems to involve an extremely slow metabolic and proliferation rate [28]. In persister cells, most biological processes are slowed down, yet efflux pump components, including *acrA, acrB,* and *tolC*, are reported to be highly expressed in persister cells and therefore have increased efflux activity, which increases their tolerance to antibiotics [29]. Recently, El Meouche and Dunlop [30] showed that increased expression of the RND efflux pump AcrAB-TolC in *Escherichia coli* resulted in lower expression of the DNA mismatch repair gene *mutS*. *MutS*-deficient cells have a mutator phenotype because they are defective in mismatch repair (MMR) and the very short patch (VSP) repair system (which removes T–G mismatches created by the deamination of 5-methylcytosine to thymine) [31]. Because MutS suppresses RecA-mediated strand transfer, *mutS*-deficient cells also have an increased recombination in interspecies crosses [31]. This finding adds another layer to the role of efflux pumps because their upregulation enables *E. coli* to survive antibiotic treatment by the “conventional” route by pumping out the antibiotics, and simultaneously increase the overall mutation rate, promoting subsequent acquisition of mutations in antibiotic target genes. Future studies will show whether this phenomenon is specific to *E. coli* or also applies to other bacterial species. It is attractive to speculate that upregulation of the RND efflux pump in *P. aeruginosa* also results in a mutator phenotype, which in part could explain the “success” of this opportunistic pathogen in being able to adapt to the harsh environment in the lungs of cystic fibrosis patients.

## 6. Inhibitors of MDR Efflux Pumps

Efflux pump inhibitors are likely to resensitize bacteria to a given antibiotic or even reverse the MDR phenotype. Thus, as expected, efflux pump inhibitors are able to prevent the transition from low-level fluoroquinolone resistance to clinical resistance in the pathogenic bacteria *Staphylococcus aureus* [32] and *P. aeruginosa* [33]. High-level resistance to fluoroquinolones is mediated by specific point mutations in the targets of this drug (gyrase and topoisomerase IV); however, in both bacteria, efflux pumps facilitate intrinsic resistance to fluoroquinolones, by the multidrug efflux MFS transporter NorA in *S. aureus* [34] and by members of the RND efflux pump family in *P. aeruginosa* [35]. Here, treating *S. aureus* with the efflux pump inhibitor reserpine or *P. aeruginosa* with the efflux pump inhibitor phenylalanine arginyl β-naphthylamide (PAβN) suppressed the in vitro emergence of high-level fluoroquinolone resistance in both cases [32,33]. Although numerous efflux pump inhibitors have been developed in recent years, none have been used in clinical applications due to side effects and in vivo toxicity [36]. However, Zimmermann et al. [37] identified nilotinib, by screening compounds already approved for clinical use, as an inhibitor of the *S. aureus* NorA, an MFS family efflux pump. The combinatorial treatment of nilotinib and the fluoroquinolone ciprofloxacin reduced *S. aureus* biofilm formation at clinically achievable concentrations [37]. Based on these data, efflux pump inhibitors represent an intriguing combinatory treatment option, alongside an antimicrobial agent. In addition, putative of the *E. coli*, the AcrAB-TolC efflux pump would be highly interesting in that it might not only sensitize cells to antibiotics but also restore their mutation rates/decreased persisters formation, potentially leading to an improved treatment outcome [38].

## 7. Conclusions and Perspectives

Efflux pumps are widespread in both Gram-negative and Gram-positive bacteria, because they create intrinsic tolerance to toxic compounds. This offers a great opportunity for infecting pathogens to survive antibiotic treatment, especially at infection sites, where antibiotics are difficult to administrate and the in vivo antibiotic concentrations are low. At some infection sites, efflux pump-mediated survival can facilitate the transition from low-level to clinical resistance, why this subject requires attention. One treatment strategy could be a combination therapy of efflux pump inhibitors and conventional antibiotics.

## References

[1] Davies S.C., Watson J., Fowler T., Livermore D.M. (2013). Annual Report of the Chief Medical Officer: Infections and the Rise of Antimicrobial Resistance. Lancet.

[2] WHO (2014). Antimicrobial Resistance. Global Report on Surveillance.

[3] Roope L.S.J., Smith R.D., Pouwels K.B., Buchanan J., Abel L., Eibich P., Butler C.C., Tan P.S., Walker A.S., Robotham J.V. (2019). The challenge of antimicrobial resistance: What economics can contribute. Science.

[4] Piddock L.J. (2006). Clinically relevant chromosomally encoded multidrug resistance efflux pumps in bacteria. Clin. Microbiol. Rev..

[5] Evans K., Passador L., Srikumar R., Tsang E., Nezezon J., Poole K. (1998). Influence of the MexAB-OprM multidrug efflux system on quorum sensing in Pseudomonas aeruginosa. J. Bacteriol..

[6] Anes J., McCusker M.P., Fanning S., Martins M. (2015). The ins and outs of RND efflux pumps in Escherichia coli. Front. Microbiol..

[7] Alav I., Sutton J.M., Rahman K.M. (2018). Role of bacterial efflux pumps in biofilm formation. J. Antimicrob. Chemother..

[8] Kuroda T., Tsuchiya T. (2009). Multidrug efflux transporters in the MATE family. Biochim. Biophys. Acta.

[9] Tseng T.T., Gratwick K.S., Kollman J., Park D., Nies D.H., Goffeau A., Saier M.H. (1999). The RND permease superfamily: An ancient, ubiquitous and diverse family that includes human disease and development proteins. J. Mol. Microbiol. Biotechnol..

[10] Lubelski J., Konings W.N., Driessen A.J. (2007). Distribution and physiology of ABC-type transporters contributing to multidrug resistance in bacteria. Microbiol. Mol. Biol. Rev..

[11] Chung Y.J., Saier M.H. (2001). SMR-type multidrug resistance pumps. Curr. Opin. Drug Discov. Dev..

[12] Hassan K.A., Jackson S.M., Penesyan A., Patching S.G., Tetu S.G., Eijkelkamp B.A., Brown M.H., Henderson P.J., Paulsen I.T. (2013). Transcriptomic and biochemical analyses identify a family of chlorhexidine efflux proteins. Proc. Natl. Acad. Sci. USA.

[13] Law C.J., Maloney P.C., Wang D.N. (2008). Ins and outs of major facilitator superfamily antiporters. Annu. Rev. Microbiol..

[14] Nikaido H. (2011). Structure and mechanism of RND-type multidrug efflux pumps. Adv. Enzymol. Relat. Areas Mol. Biol..

[15] Hassan K.A., Liu Q., Elbourne L.D.H., Ahmad I., Sharples D., Naidu V., Chan C.L., Li L., Harborne S.P.D., Pokhrel A. (2018). Pacing across the membrane: The novel PACE family of efflux pumps is widespread in Gram-negative pathogens. Res. Microbiol..

[16] WHO (2017). Global Priority List of Antibiotic-Resistant Bacteria to Guide Research, Discovery, and Development of New Antibiotics.

[17] Lamut A., Peterlin Masic L., Kikelj D., Tomasic T. (2019). Efflux pump inhibitors of clinically relevant multidrug resistant bacteria. Med. Res. Rev..

[18] Fernandez L., Hancock R.E. (2012). Adaptive and mutational resistance: Role of porins and efflux pumps in drug resistance. Clin. Microbiol. Rev..

[19] Gould V.C., Okazaki A., Avison M.B. (2013). Coordinate hyperproduction of SmeZ and SmeJK efflux pumps extends drug resistance in Stenotrophomonas maltophilia. Antimicrob. Agents Chemother..

[20] Llanes C., Hocquet D., Vogne C., Benali-Baitich D., Neuwirth C., Plesiat P. (2004). Clinical strains of Pseudomonas aeruginosa overproducing MexAB-OprM and MexXY efflux pumps simultaneously. Antimicrob. Agents Chemother..

[21] Andersson D.I., Balaban N.Q., Baquero F., Courvalin P., Glaser P., Gophna U., Kishony R., Molin S., Tonjum T. (2020). Antibiotic resistance: Turning evolutionary principles into clinical reality. FEMS Microbiol. Rev..

[22] Frimodt-Moller J., Rossi E., Haagensen J.A.J., Falcone M., Molin S., Johansen H.K. (2018). Mutations causing low level antibiotic resistance ensure bacterial survival in antibiotic-treated hosts. Sci. Rep..

[23] Marvig R.L., Sommer L.M., Molin S., Johansen H.K. (2015). Convergent evolution and adaptation of Pseudomonas aeruginosa within patients with cystic fibrosis. Nat. Genet..

[24] Pule C.M., Sampson S.L., Warren R.M., Black P.A., van Helden P.D., Victor T.C., Louw G.E. (2016). Efflux pump inhibitors: Targeting mycobacterial efflux systems to enhance TB therapy. J. Antimicrob. Chemother..

[25] Dewachter L., Fauvart M., Michiels J. (2019). Bacterial Heterogeneity and Antibiotic Survival: Understanding and Combatting Persistence and Heteroresistance. Mol. Cell.

[26] Baquero F. (2001). Low-level antibacterial resistance: A gateway to clinical resistance. Drug Resist. Updates.

[27] Pacheco J.O., Alvarez-Ortega C., Rico M.A., Martinez J.L. (2017). Metabolic Compensation of Fitness Costs Is a General Outcome for Antibiotic-Resistant Pseudomonas aeruginosa Mutants Overexpressing Efflux Pumps. mBio.

[28] Lewis K. (2007). Persister cells, dormancy and infectious disease. Nat. Rev. Microbiol..

[29] Pu Y., Zhao Z., Li Y., Zou J., Ma Q., Zhao Y., Ke Y., Zhu Y., Chen H., Baker M.A.B. (2016). Enhanced Efflux Activity Facilitates Drug Tolerance in Dormant Bacterial Cells. Mol. Cell.

[30] El Meouche I., Dunlop M.J. (2018). Heterogeneity in efflux pump expression predisposes antibiotic-resistant cells to mutation. Science.

[31] Marinus M.G. (2012). DNA Mismatch Repair. EcoSal Plus.

[32] Markham P.N., Westhaus E., Klyachko K., Johnson M.E., Neyfakh A.A. (1999). Multiple novel inhibitors of the NorA multidrug transporter of Staphylococcus aureus. Antimicrob. Agents Chemother..

[33] Lomovskaya O., Warren M.S., Lee A., Galazzo J., Fronko R., Lee M., Blais J., Cho D., Chamberland S., Renau T. (2001). Identification and characterization of inhibitors of multidrug resistance efflux pumps in Pseudomonas aeruginosa: Novel agents for combination therapy. Antimicrob. Agents Chemother..

[34] Neyfakh A.A. (1992). The Multidrug Efflux Transporter of Bacillus-Subtilis Is a Structural and Functional Homolog of the Staphylococcus Nora Protein. Antimicrob. Agents Chemother..

[35] Lister P.D., Wolter D.J., Hanson N.D. (2009). Antibacterial-Resistant Pseudomonas aeruginosa: Clinical Impact and Complex Regulation of Chromosomally Encoded Resistance Mechanisms. Clin. Microbiol. Rev..

[36] Spengler G., Kincses A., Gajdacs M., Amaral L. (2017). New Roads Leading to Old Destinations: Efflux Pumps as Targets to Reverse Multidrug Resistance in Bacteria. Molecules.

[37] Zimmermann S., Klinger-Strobel M., Bohnert J.A., Wendler S., Rodel J., Pletz M.W., Loffler B., Tuchscherr L. (2019). Clinically Approved Drugs Inhibit the Staphylococcus aureus Multidrug NorA Efflux Pump and Reduce Biofilm Formation. Front. Microbiol..

[38] Frimodt-Moller J., Lobner-Olesen A. (2019). Efflux-Pump Upregulation: From Tolerance to High-level Antibiotic Resistance?. Trends Microbiol..

